# Supplementary material for: Differences in Willingness to Undergo BRCA1/2 Testing and Risk Reducing Surgery among the General Public, Cancer Patients, and Healthcare Professionals: A Large Population-Based Survey
Source: J Pers Med. 2022 May 18;12(5):818. doi: 10.3390/jpm12050818 (PMC9147340; doi:10.3390/jpm12050818)
Supplement: Supplementary file 1 [file jpm-12-00818-s001.zip › jpm-1719339-supplementary.pdf]

Supplementary Materials

Scenario 1. Intention to undergo BRCA genetic testing

BRCA genetic test is a useful tool for estimating the possibility of developing breast cancer for individuals who have one or more family members that have been diagnosed with breast cancer. This situation was brought to public awareness by Angelina Jolie, a famous Hollywood actress, who chose to undergo testing. In general, the test is recommended to individuals if one or more of their family members are diagnosed with breast, ovarian, or prostate cancer, which indicates that they are at high risk of developing cancer. In addition, 15% of people who underwent the *BRCA* genetic test were found to harbor the mutation.

At age 37, Mrs. Kim, with a lump in her right breast, was tested and diagnosed with breast cancer. After explaining the *BRCA* genetic test, her doctor suggested that she undergo the test to determine whether the cancer was caused by an inherited mutation.

Question: If you were Mrs. Kim, would you undergo the *BRCA* genetic test?

Scenario 2. Intention to undergo prophylactic salpingo-oophorectomy or prophylactic mastectomy

Among the total population who underwent the *BRCA* genetic test, 15% harbored the *BRCA* mutation.

The test result for Mrs. Park revealed the presence of a *BRCA* mutation, which indicated that she was at high risk of developing breast cancer; she was advised to undergo a regular screening test or counseling with a doctor regarding undergoing preventive prophylactic salpingo-oophorectomy or prophylactic mastectomy.

Question: If you were Mrs. Park, would you undergo prophylactic salpingo-oophorectomy for the purpose of prevention?

Table S1. Multivariate logistic regression analysis of variables associated with intent to undergo *BRCA1/2* testing

Variables	All (n = 3,444)	General public (n = 1,496)	Cancer patients (n = 1,500)	Clinicians (n = 108)	Researchers (n = 340)
Group					
General public	1				
Cancer patients	**1.94 (1.62–2.31)**				
Clinicians	**3.60 (1.77–7.32)**				
Researchers	**4.66 (2.97–7.32)**				
Gender					
Female	1	1	1		
Male	**0.71 (0.61–0.83)**	**0.71 (0.58–0.87)**	**0.71 (0.56–0.90)**		
Age, years					
20–39	1		1		
40–49	0.92 (0.74–1.14)		0.60 (0.33–1.08)		
≥50	0.81 (0.66–1.00)		**0.46 (0.26–0.80)**		
Education Level					
≤High school	1		1		
College graduate	1.09 (0.92–1.29)		1.17 (0.88–1.54)		1
Graduate degree	1.36 (0.87–2.15)		0.74 (0.39–1.40)		**2.24 (1.09–4.61)**
Household monthly income					
≤USD 3000	1		1		
USD 3000–4999	**1.30 (1.09–1.56)**		**1.61 (1.25–2.07)**		
≥USD 5000	**1.47 (1.09–1.98)**		1.54 (0.95–2.49)		
Living area					
Metro					
Non-metro					
Perceived health status					
Excellent/Good			1		
Fair/Poor/Very Poor			0.81 (0.64–1.02)		

Adjusted OR (95% CI). OR = odds ratio; 95% CI = 95% confidence interval; multivariate adjustment comparisons were performed using logistic regression (backward procedure, Wald test). Boldface indicates statistical significance ($p < 0.05$).

Table S2. Multivariate logistic regression analysis of variables associated with intent to undergo risk-reducing mastectomy

Variables	All (n = 3,444)	General public (n = 1,496)	Cancer patients (n = 1,500)	Clinicians (n = 108)	Researchers (n = 340)
Group					
General public					
Cancer patients					
Clinicians					
Researchers					
Gender					
Female	1	1			1
Male	**0.76 (0.65–0.89)**	**0.57 (0.45–0.72)**			**2.02 (1.23–3.30)**
Age, years					
20–39					
40–49					
≥50					
Education Level					
≤High school	1				
College graduate	**1.20 (1.01–1.42)**				
Graduate degree	1.17 (0.88–1.56)				
Household monthly income					
≤USD 3000	1		1		
USD 3000–4999	1.12 (0.94–1.35)		1.13 (0.88–1.44)	1	
≥USD 5000	1.20 (0.91–1.57)		**1.87 (1.24–2.81)**	**0.34 (0.12–0.96)**	
Living area					
Metro					
Non-metro					
Perceived health status					
Excellent/Good					
Fair/Poor/Very Poor					

Adjusted OR (95% CI. OR = odds ratio; 95% CI = 95% confidence interval; multivariate adjustment comparisons were performed using logistic regression(backward procedure, Wald test). Boldface indicates statistical significance ($p < 0.05$).
